# Comparative changes in emergency department patient attendance at the pediatric emergency hospital during the first wave between 2019 and 2020

**DOI:** 10.17843/rpmesp.2022.393.11245

**Published:** 2022-09-30

**Authors:** Ángela Stramandinoli, Julio Alvarado-Zúñiga, Germán F. Alvarado, Gabriela Egúsquiza-Zuzunaga, Nilton Yhuri Carreazo

**Affiliations:** 1 Medical School, Universidad Peruana de Ciencias Aplicadas, Lima, Peru. Universidad Peruana de Ciencias Aplicadas Medical School Universidad Peruana de Ciencias Aplicadas Lima Peru; 2 Pediatric Emergency Hospital, Lima, Peru. Pediatric Emergency Hospital Lima Peru

**Keywords:** Pediatrics, Emergency care, Emergency Service, Hospital, Pandemics, Delivery of Health Care, Peru, COVID-19

## Abstract

The COVID-19 pandemic changed the pattern of patient attendance at healthcare facilities. We describe the comparative changes in the attendance of pediatric patients at the Emergency Department of the Pediatric Emergency Hospital of Lima, Peru, between epidemiological weeks 10 to 48 of 2019 and 2020. Sociodemographic data, patient condition, type of insurance and diagnosis grouped according to ICD-10 code were analyzed. We found a 48.2% reduction in the number of visits in 2020 when compared to 2019, and a five-fold increase in the number of visits from localities other than Metropolitan Lima. Likewise, the probability of diagnosis of “infectious and parasitic diseases” increased by 27% and the probability of diagnosis of “diseases of the respiratory system” decreased by 61%.

## INTRODUCTION

In late 2019, an outbreak of pneumonia, etiology unknown, began in the Wuhan locality of China. The causative agent was identified on January 7, 2020, as a new type of coronavirus (SARS-CoV-2) ^1^ ^,^ ^2^. Subsequently, on January 30, the World Health Organization (WHO) declared this situation a “public health emergency of international concern” ^3^, and finally enacted pandemic status on March 11, 2020 ^4^.

In Peru, the first case of COVID-19 was confirmed on March 6, 2020. National quarantine was declared on March 16 and lasted until June 30 ^5^ ^,^ ^6^. According to the National Center for Epidemiology, Prevention and Disease Control (CDC-Peru), the first wave occurred between epidemiological weeks 10 and 48 ^7^. The general population was advised not to go to hospitals, which delayed seeking medical attention ^8^. Likewise, Mejía *et al*. reported that in Peru, users exposed to news about the pandemic through the media experienced an increase in the perception of fear ^9^.

In Italy, Liguoro *et al*. found a reduction in emergency department attendance when comparing 2018, 2019 and 2020 attendance ^10^. Likewise, there was a 70% decrease in pediatric patient care in the emergency department in a university hospital in New Jersey, USA; particularly in March, compared to the years 2018 to 2020 ^11^.

Due to the pandemic, some studies have found a significant decrease in several diagnoses, mainly those related to respiratory diseases, trauma, and infectious diseases ^12^ ^,^ ^13^.

Although there is worldwide evidence of a decrease in the number of visits to health centers, there are few national studies on the pediatric population. Therefore, this research aims to describe the characteristics of care in the emergency department of the Hospital de Emergencias Pediátricas (HEP) during the COVID-19 pandemic and compare the change in the visits of pediatric patients during epidemiological weeks 10 to 48 of the years 2019 and 2020.

**KEY MESSAGES**

Motivation for the study: national studies on the epidemiological behavior of emergency department attendance in the pediatric population during the first wave of COVID-19 are scarce.

Main findings: we found a decrease in the number of visits of pediatric patients to the emergency department in 2020, as well as a decrease of respiratory diseases. The increase in infectious diseases and trauma, poisoning and other external causes stands out.

Implications: the findings of this study will serve to improve the availability of resources in healthcare centers, according to the recognition of the distribution variation of diseases in a pandemic.

## THE STUDY

### Study design

We conducted a retrospective cross-sectional study, based on the analysis of a secondary database of patients who visited the emergency department of the HEP located in Lima, Peru during 2019 and 2020.

### Population, sample, and sampling

The population consisted of 41,470 pediatric patients who visited the emergency department of the HEP between epidemiological weeks 10 to 48 of the years 2019 and 2020. We used a census from which we excluded patients who were of legal age, those who voluntarily withdrew during their stay, and records with incomplete data.

### Procedures

The database was constructed by the HEP from the information in the medical records. The corresponding permissions were obtained from the Teaching and Research Support Office of the hospital. The data were exported to the Excel 2019 program to be anonymized. Information regarding consecutive re-evaluation of a single patient was eliminated. Subsequently, exclusion criteria were applied. Finally, the database was exported to the statistical program STATA version 15 (StataCorp, College Station, TX) for analysis.

### Variables

The main variables were: number of attendants, and the month and week of admission to the emergency department of the HEP. Other variables were included to characterize the demographics of the population, such as age, sex, place of origin, area of origin within Metropolitan Lima, type of insurance, condition and destination of the patient and ICD-10 diagnostic group.

### Statistical analysis

We used absolute frequencies and proportions to describe the categorical variables. For the percentage calculation, the number of diagnoses grouped by ICD-10 was divided by the total number of patients seen during the year and multiplied by 100. Subsequently, the percentage difference between the years 2020 and 2019 was determined. The chi-square test was used during the bivariate analysis. The crude prevalence ratio (PR), and 95% confidence interval, was calculated for each diagnostic category as the proportion of patients seen during the pandemic period divided by the proportion of patients seen during the pre-pandemic period.

### Ethical Aspects

This study was approved by the Ethics and Research Subcommittee (CEI) of the Universidad Peruana de Ciencias Aplicadas (UPC). Since our study was based on the analysis of a database of medical records from a public hospital, the data that allowed recognition of the patients were anonymized. No records were discriminated by sex, age, place of origin or socioeconomic status. Informed consent was not required because the information was obtained from a database. The code of the Health Research Projects Registry (PRISA) of the Peruvian National Institute of Health was EI00000002568.

## FINDINGS

**Figure 1:**
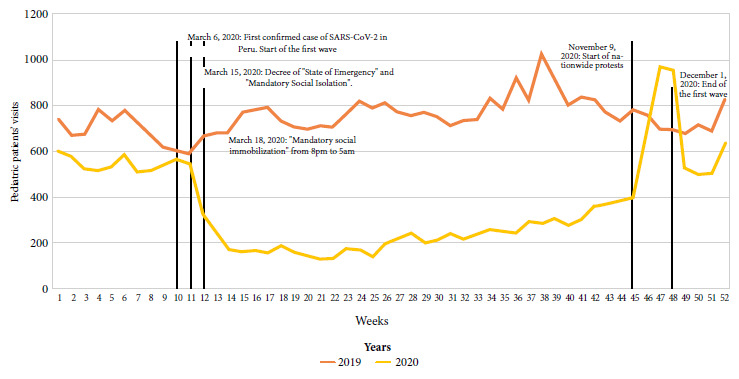
Pediatric patient attendance by weeks in the emergency department of the Pediatric Emergency Hospital between 2019 and 2020 (n=57,845).

At the beginning of 2020, the average number of patients decreased (Figure 1). After the state of emergency and mandatory social isolation were declared, the HEP emergency department showed a decrease in the number of patients, from 669 in 2019 to 326 in 2020. From week 25 onwards, there was a gradual increase in the number of patients. In week 45, we found an increase with its maximum peak in week 47 with 970 visits in 2020.

Table 1 shows an average decrease of 10.2% in the number of visits by preschoolers in 2020. Regarding the place of origin. we found a five-fold increase in the number of patients coming from places other than Metropolitan Lima. According to the type of insurance, there was a proportional increase of 3.3% of patients with Comprehensive Health Insurance (SIS) and a proportional decrease of the same figure of patients without insurance.

**Table 1:** Demographic characteristics of pediatric patients who visited the Emergency Department of the Pediatric Emergency Hospital during the first wave in the between 2019 and 2020 (n=41,470).

Characteristic	Pre-COVID (2019) (n=29,736)		COVID (2020) (n=11,734)		p-value
	n	%	n	%	
Sex					0.012
Female	13,031	43.8	5302	45.2	
Male	16,70	56.2	6432	54.8	
Age group					<0.001
Infants (<12 months)	1755	5.9	1319	11.2	
Preschool children (1 - 5 years)	19,958	67.1	6685	56.9	
Schoolchildren (6 - 11 years)	6284	21.1	2660	22.7	
Adolescents (12 - 17 years)	1739	5.9	1070	9.1	
Place of origin					<0.001
Metropolitan Lima	28,772	96.8	9737	82.9	
Other regions	964	3.2	1997	17.0	
Zone of Lima					0.553
North	1227	4.3	495	4.4	
Center	12,036	41.8	4783	42.4	
South	1710	5.9	679	6.0	
West	13,799	47.9	5319		
Type of insurance					<0.001
SIS	24,443	82.2	10,028	85.5	
Private	44	0.2	25	0.2	
Social Service	35	0.1	6	0.0	
No insurance	5214	17.5	1675	14.3	

SIS: Comprehensive Health Insurance.

Social service: hospital worker + special support

Table 2 shows a 7.5% increase in new patients and a 10% average decrease in continuing patients in 2020 compared to 2019. Likewise, we found a 4.1% decrease in the number of discharges from the HEP emergency department in 2020.

**Table 2:** Admission status and destination of pediatric patients who visited the Emergency Department of the Pediatric Emergency Hospital during the first wave in between 2019 and 2020 (n=41,470).

Characteristic	Pre-COVID (2019) (n= 29,736)		COVID (2020) (n= 11,734)		p-value
	n	%	n	%	
Status at admission					<0.001
New	9608	32.3	4668	39.8	
Re-evaluated	8261	27.8	3563	30.4	
Continuing	11,867	39.9	3503	29.9	
Destination					<0.001
Discharge	23,801	80.0	8913	75.9	
Discharge + telemonitoring	0	0	288	2.5	
Hospitalization	671	2.3	538	4.6	
Re-evaluation	2620	8.8	1252	10.7	
Operating room	227	0.8	144	1.2	
Observation room	2324	7.8	534	4.6	
Referral	88	0.3	62	0.5	
Deceased	5	0.0	3	0.0	

Re-evaluation: re-evaluation + interconsultation + oral rehydration room + bronchial asthma room

When comparing diagnoses by ICD-10 code, we found that in 2020 there was 27% higher probability of diagnosis of “A00-B99: infectious and parasitic diseases” compared to 2019; this is shown in Table 3. In the case of “J00-J99: diseases of the respiratory system” we found that in 2020 there was 61% lower probability compared to 2019. At the same time, we observed 80% higher probability of the diagnosis of “S00-T98: trauma, poisoning and some other consequences of external cause” in 2020 compared to 2019. Likewise, there was a 32% decrease in the diagnostic probability of “K00-K93: diseases of the digestive system” in 2020 compared to 2019. Finally, an average of 21 cases per week was found during 2020 for code U00-U99, which included patients with COVID-19 diagnosis.

**Table 3:** Diagnosis by ICD-10 code of pediatric patients who visited the Emergency Department of the Pediatric Emergency Hospital during the first wave between 2019 and 2020 (n=76 254).

ICD-10 Group Diagnosis	Pre-COVID (2019) (n = 55,801)		COVID (2020) (n = 20,453)		Percentage Difference %	Crude PR (95%CI)
	n	%	n	%		
R00- R99: Symptoms, signs, and abnormal clinical and laboratory findings, not elsewhere classified	3,902	7.0	5,350	26.2	19.2	3.74 (3.60 - 3.88)
U00-U99: Codes for special purposes	0	0	790	3.9	3.9	-
U00-U99: Códigos para situaciones especiales	1,118	2.0	1,145	5.6	3.6	2.79 (2.57 - 3.02)
C00-D48: Neoplasms	29	0.1	53	0.3	0.2	4.98 (3.17 - 7.83)
Q00-Q99: Congenital malformations, deformations, and chromosomal abnormalities	217	0.4	226	1.1	0.7	2.84 (2.36 - 3.42)
F00-F99: Mental and behavioural disorders	54	0.1	63	0.3	0.2	3.18 (2.21 - 4.57)
Z00-Z99: Factors influencing health status and contact with health services.	179	0.3	188	0.9	0.6	2.86 (2.33 - 3.51)
O00-O99: Pregnancy, childbirth, and puerperium	1	0.0	2	0.0	0.0	5.45 (0.49 - 60.17)
D50-D89: Diseases of the blood and hematopoietic organs and other disorders affecting the mechanism of immunity.	44	0.1	23	0.1	0.0	1.42 (0.86 - 2.36)
P00-P96: Certain conditions originating in the perinatal period	67	0.1	37	0.2	0.1	1.50 (1 - 2.25)
V00-Y98: External causes of morbidity and mortality	415	0.7	384	1.9	1.2	2.52 (2.19 - 2.89)
G00-G99: Nervous system diseases	206	0.4	158	0.8	0.4	2.09 (1.70 - 2.57)
M00-M99: Musculoskeletal system and connective tissue diseases	201	0.4	139	0.7	0.3	1.88 (1.52 - 2.34)
H00-H59: Diseases of the eye and adnexa	92	0.2	26	0.1	-0.1	0.77 (0.49 - 1.19)
L00-L99: Diseases of the skin and subcutaneous tissue	1437	2.6	1 165	5.7	3.1	2.21 (2.05 - 2.38)
I00-I99: Diseases of the circulatory system	536	1.0	97	0.5	-0.5	0.49 (0.39 - 0.61)
E00-E90: Endocrine, nutritional, and metabolic diseases	899	1.6	299	1.5	-0.1	0.90 (0.79 - 1.03)
H60-H95: Diseases of the ear and mastoid process	811	1.5	149	0.7	-0.8	0.50 (0.42 - 0.59)
S00-T98: Trauma poisoning and certain other consequences of external causes	3847	6.9	2551	12.5	5.6	1.80 (1.72 - 1.90)
A00-B99: Certain infectious and parasitic diseases	9956	17.9	4646	22.8	4.9	1.27 (1.23 - 1.31)
K00-K93: Diseases of the digestive system	9725	17.4	2426	11.9	-5.5	0.68 (0.65 - 0.71)
J00-J99: Diseases of the respiratory system	10,488	18.8	1534	7.5	-11.3	0.39 (0.37 - 0.41)

PR: prevalence ratio

## DISCUSSION

We observed a reduction of 60.5% in the number of visits to the emergency department of the HEP after the first wave began in the national territory, with a greater decrease in the number of preschool patients (10.2%). There was also an increase in the number of patients who came from regions other than Metropolitan Lima (13.8%). At the same time, there was a 61% decrease in the probability of suffering from “respiratory system diseases” and an 80% increase in the diagnostic group of “trauma, poisoning and some other consequences of external causes” when compared to 2019.

The reduction in cases is similar to what was reported by Jang *et al*. who found a 62% decrease in pediatric patients’ attendance at the emergency department in a Korean hospital in 2020 ^14^. This result may reflect a decrease in the need to use the emergency department or be related to the delay of serious diagnoses; detailed in the reports by Levine and Isba ^15^ ^,^ ^16^.

As mentioned, there is concern about the reduction in the number of pediatric patient visits. Pepper *et al*. reported a 48% decrease in the most urgent care in the pediatric emergency department ^11^. In addition, Pines *et al*. detailed a decrease in cases of appendicitis, sepsis, and intussusception of 19%, 49%, and 42%, respectively, in 2020 compared with 2019 ^17^.

In 2020 there was a gradual increase in the number of visits to the emergency department of the HEP. This would be related to the need not to comply with quarantine, since total isolation is not feasible for the average family. An increase in the number of cases was observed from week 45 onwards, which coincides with the social mobilizations that took place in the country ^18^, which could have increased the transmission of diseases.

The increase in the number of cases from outside Metropolitan Lima is possibly explained by a greater acceptance of referrals from health facilities with a lower resolution capacity. Likewise, the decrease in the number of uninsured patients is proportional to the increase in the number of patients who are affiliated to the SIS, which is designed to protect the most vulnerable sectors of the population. In 2020 there was an increase in new and re-evaluated patients compared to the previous year; however, there was a reduction in the number of continuing patients. One possible explanation is that these patients did not attend their consultations due to fear of COVID-19 infection.

Regarding diagnoses, the most striking change is the decrease in “respiratory system diseases” in 2020, explained by the quarantine and the use of mandatory masks. In Italy, Brisca *et al*. reported similar findings with a reduction of cases of respiratory diseases by 10.8% ^12^. Similarly, Raucci *et al*. found a 63% decrease in respiratory diseases due to restrictive measures and low exposure to inhaled allergens ^19^.

In 2020, diagnoses of “infectious and parasitic diseases” increased, possibly due to contagion through fomites, poor hand washing or inefficient storage of food for later consumption. A study conducted in Italy described an increase of 1.1% in diseases associated with the digestive system ^19^. Likewise, regarding “trauma and poisoning,” the literature refers to a decrease in the number of cases, since outdoor activities were restricted ^2^ ^,^ ^19^ ^,^ ^20^. However, an increase in this type of diagnoses was found, which could be related to domestic abuse and neglect during quarantine ^20^.

Our has several limitations. In first place, by analyzing a single health facility, the results cannot be applied to the Peruvian pediatric population. In second place, the diagnoses were based on the criteria of the physician who completed the medical record from which the data were obtained for the hospital database. Finally, by using a secondary database, the analysis can only be carried out with the available data. For example, it was not possible to inquire about the reasons for patient care or to assess the severity of the disease on arrival at the emergency department, due to the lack of triage information.

It is concluded that there was a decrease in the number of emergency department visits to the HEP Emergency Department in 2020 compared to 2019. Likewise, an increase in patients from regions other than Metropolitan Lima was found. In addition to these findings, there was a decrease in “respiratory system diseases” and an increase in “trauma and poisoning” cases.

Due to these findings, the authors recommend analyzing the factors that may have influenced the increase in trauma cases in the emergency department of the HEP. This recommendation is based on the various programs against domestic violence such as the AURORA National Program, which has various strategies to prevent this problem. Similarly, several multicenter studies should be conducted in order to apply the results to the Peruvian pediatric population in 2019 and 2020.

## References

[1] Zheng J (2020). SARS-coV-2 An emerging coronavirus that causes a global threat. Int J Biol Sci.

[2] Serra A (2020). Infección respiratoria aguda por COVID-19 una amenaza evidente. Rev Habanera Ciencias Medicas.

[3] Rothan HA, Byrareddy SN (2020). The epidemeology and pathogensis of coronavirus (Covid-19) outbreak. J Autoimmun.

[4] World Health Organization (2020). WHO Director-General's opening remarks at the media briefing on COVID-19 - 11 March 2020.

[5] Huamaní C, Timaná-Ruiz R, Pinedo J, Pérez J, Vásquez L (2020). Condiciones estimadas para controlar la pandemia de COVID-19 en escenarios de pre y poscuarentena en el Perú. Rev Peru Med Exp Salud Publica.

[6] Rodriguez-Morales AJ, Sánchez-Duque JA, Hernández Botero S, Pérez-Díaz CE, Villamil-Gómez WE, Méndez CA (2020). Preparación y control de la enfermedad por coronavirus 2019 (COVID-19) en América Latina. Acta Med Peru.

[7] Ministerio de Salud (2022). Situación del COVID-19. Perú. 2020 / 2021.

[8] Ciacchini B, Tonioli F, Marciano C, Faticato MG, Borali E, Pini Prato A (2020). Reluctance to seek pediatric care during the COVID-19 pandemic and the risks of delayed diagnosis. Ital J Pediatr.

[9] Mejía C, Rodriguez-Alarcón J, l Garay-Rios, Enriquez-Anco M, Moreno A, Huaytan-Rojas K (2020). Percepción de miedo o exageración que transmiten los medios de comunicación en la población peruana durante la pandemia de la COVID-19. Rev Cubana Invest Bioméd.

[10] Liguoro I, Pilotto C, Vergine M, Pusiol A, Vidal E, Cogo P (2021). The impact of COVID-19 on a tertiary care pediatric emergency department. Eur J Pediatr.

[11] Pepper M, Leva E, Trivedy P, Luckey J, Baker M (2020). Analysis of pediatric emergency department patient volume trends during the covid 19 pandemic. Med.

[12] Brisca G, Vagellli G, Tagliarini G, Rotulo A, Pirlo D, Romanengo M (2020). The impact of COVID-19 lockdown on children with medical complexity in pediatric emergency department. Am J Emerg Med.

[13] Rotulo G, Percivale B, Molteni M, Naim A, Brisca G, Piccotti E (2021). The impact of COVID-19 on infectious diseases epidemiology The experience of a tertiary Italian Pediatric Emergency department. Em J Emerg Med.

[14] Jang K, Ahn J, Choi H, Lee S, Kim D, Lee D (2021). Pediatric emergency department utilization and coronavirus disease in Daegu, Korea. J Korean Med Sci.

[15] Levine D, Fraymovich S, Shari L, Platt M (2020). Where are all the children gone In New York City. Ann Emerg Med.

[16] Isba R, Edge R, Auerbach M, Cicero M, Jenner R, Setzer E (2020). Transatlantic declines in pediatrics emergency admissions. Pediatr Emerg Care.

[17] Pines J, Zocchi M, Black B, Carlson J, Celedon P, Moghtaderi A (2021). Characterizing pediatric emergency department visits during the COVID-19 pandemic. Am J Emerg Med Pediatrics.

[18] Murguerza M, Gonzales-García C (2022). Generación del Bicentenario movimientos juveniles contra el expresidente Merino. Universitas-XXI.

[19] Raucci U, Musolino A, Lallo D, Piga S, Barbieri M, Pisani M (2021). Impact of the COVID-19 pandemic on the emergency department of a tertiary children's hospital. Ital J Pediatr.

[20] Dopfer C, Watzke M, Scharff A, Mueller F, Dressler F, Baumann U (2020). COVID-19 related reduction in pediatric emergency healthcare utilization - a concerning trend. BMC Pediatr.

