# Supplementary material for: Comparative changes in emergency department patient attendance at the pediatric emergency hospital during the first wave between 2019 and 2020
Source: Rev Peru Med Exp Salud Publica. 2022 Sep 30;39(3):345–51. doi: 10.17843/rpmesp.2022.393.11245 (PMC11397767; doi:10.17843/rpmesp.2022.393.11245)
Supplement: Translation

# Translation: Cambios comparativos en la asistencia de pacientes al servicio de emergencia durante la primera ola en el Hospital de Emergencias Pediátricas entre el 2019 y 2020

**Authors:** Ángela Stramandinoli, Julio Alvarado-Zúñiga, Germán F. Alvarado, Gabriela Egúsquiza-Zuzunaga, Nilton Yhuri Carreazo

**Affiliations:** 1 Escuela de Medicina, Universidad Peruana de Ciencias Aplicadas, Lima, Perú.; 2 Hospital de Emergencias Pediátricas, Lima, Perú.

**Keywords:** Pediatría, Atención de Emergencias, Servicio de Urgencia en Hospital, Pandemia, Prestación de Atención de Salud, Perú, COVID-19

## Abstract

La pandemia de la COVID-19 cambió el patrón de asistencia de los pacientes a los centros de salud. Se describen los cambios comparativos en la asistencia de pacientes pediátricos que acudieron al servicio de Emergencia del Hospital de emergencias Pediátricas de Lima, Perú, entre las semanas epidemiológicas 10 a 48 del 2019 y 2020. Se analizaron datos sociodemográficos, condición del paciente, tipo de seguro y diagnóstico agrupado según código CIE-10. Se encontró una reducción del 48,2% en el número de atenciones en el 2020 con respecto al 2019 y un aumento de cinco veces el número de atenciones procedentes de localidades diferentes de Lima Metropolitana. Asimismo, se encontró un aumento del 27% en la probabilidad del diagnóstico de «enfermedades infecciosas y parasitarias» y una disminución del 61% en la probabilidad de diagnóstico de «enfermedades del sistema respiratorio».

## INTRODUCCIÓN

A finales del 2019 empezó un brote de neumonías, de etiología desconocida, en la localidad de Wuhan, China. El agente causal fue reconocido el 7 de enero del 2020, identificándose un nuevo tipo de coronavirus (SARS-CoV-2) ^1^ ^,^ ^2^. Posteriormente, el 30 de enero, la Organización Mundial de la Salud (OMS) declaró esta situación como una «emergencia de salud pública de importancia internacional» ^3^, para, finalmente, promulgar el estado de pandemia el 11 de marzo del 2020 ^4^. 

En Perú, el primer caso de COVID-19 se confirmó el 6 de marzo del 2020. El 16 de marzo se decretó la cuarentena nacional que duro hasta el 30 de junio ^5^ ^,^ ^6^. Según el Centro Nacional de Epidemiología, Prevención y Control de Enfermedades (CDC-Perú) la primera ola se consideró entre la semana epidemiológica 10 y 48 ^7^. Se desaconsejó a la población en general acudir a los hospitales; lo que retrasó la búsqueda de atención médica ^8^. Asimismo, Mejía *et al*. refirieron que en Perú los usuarios expuestos a noticias sobre la pandemia, a través de medios de comunicación experimentaron un aumento de la percepción del miedo ^9^.

En Italia, Liguoro *et al*. encontraron una reducción en la asistencia al departamento de emergencia comparando la asistencia de los años 2018, 2019 y 2020 ^10^. Asimismo, en un hospital universitario de Nueva Jersey en Estados Unidos; hubo una disminución del 70% en la atención de pacientes pediátricos en el servicio de emergencia, sobre todo en marzo, comparado con los años 2018 al 2020 ^11^.

Debido a la pandemia, algunos estudios han encontrado una disminución significativa en diversos diagnósticos, principalmente aquellos que se encuentran relacionados con enfermedades respiratorias, traumatismos y enfermedades infecciosas ^12^ ^,^ ^13^.

A pesar de existir evidencia mundial de la disminución de las atenciones en los centros de salud, existen escasos estudios nacionales realizados en población pediátrica. Por ello, esta investigación tiene el objetivo de describir las características de las atenciones en el servicio de emergencia del Hospital de Emergencias Pediátricas (HEP) durante la pandemia de la COVID-19 y comparar el cambio en la asistencia de los pacientes pediátricos durante las semanas epidemiológicas 10 a 48 de los años 2019 y 2020.

**MENSAJE CLAVE**

Motivación para realizar el estudio: existe una escasez de estudios nacionales sobre el comportamiento epidemiológico de la asistencia a los servicios de emergencia en la población pediátrica, durante la primera ola de la COVID-19.

Principales hallazgos: se demuestra una disminución en la asistencia de pacientes pediátricos al servicio de emergencias en el año 2020, al igual que en la presentación de enfermedades respiratorias. Destaca el aumento de enfermedades infecciosas y de traumatismos, envenenamientos y otras causas externas.

Implicancias: los hallazgos de este estudio servirán para mejorar la disposición de recursos en los centros de salud, según el reconocimiento de la variación en la distribución de enfermedades en situación de pandemia.

## EL ESTUDIO

### Diseño del estudio

Se realizó un estudio transversal retrospectivo, basado en el análisis de una base de datos secundaria de los pacientes atendidos en el servicio de emergencia del HEP ubicado en Lima, Perú durante los años 2019 y 2020.

### Población, muestra y muestreo

La población estuvo conformada por 41 470 pacientes pediátricos que acudieron al servicio de emergencias del HEP entre las semanas epidemiológicas 10 a 48 de los años 2019 y 2020. Se utilizó un censo del que se excluyó a pacientes mayores de edad, a los que se retiraron voluntariamente durante su estadía y los registros con datos incompletos.

### Procedimientos

La base de datos fue elaborada por el HEP a partir de la información de las historias clínicas. Se obtuvieron los permisos correspondientes de la Oficina de Apoyo a la Docencia e Investigación del hospital. Los datos fueron exportados al programa Excel 2019 para ser anonimizados y se eliminó la información consecutiva de reevaluación en un mismo paciente. Posteriormente, se aplicaron los criterios de exclusión. Por último, la base de datos fue exportada al programa estadístico STATA versión 15 (StataCorp, College Station, TX) para su análisis.

### Variables

Las variables principales fueron: el número de asistentes, el mes y la semana de admisión al servicio de emergencia del HEP. Se incluyeron otras variables para caracterizar la demografía de la población estudiada, como edad, sexo, lugar de procedencia, zona de procedencia dentro de Lima Metropolitana, tipo de seguro, condición y destino del paciente y grupo de diagnóstico CIE-10.

### Análisis estadístico

Para la descripción de las variables categóricas se usaron frecuencias absolutas y proporciones. Para el cálculo de porcentaje se utilizó el número de diagnósticos agrupados por CIE-10 sobre el número total de pacientes atendidos en el año y se multiplicó por 100. Posteriormente, se determinó la diferencia porcentual entre los años 2020 y 2019. En el análisis bivariado se utilizó la prueba de chi cuadrado. La razón de prevalencia (RP) cruda y el intervalo de confianza al 95% se calculó para cada categoría de diagnóstico como la proporción de pacientes atendidos durante el período pandémico, dividida entre la proporción de pacientes durante el período prepandémico.

### Aspectos éticos

El presente estudio tuvo la aprobación del Subcomité de Ética e Investigación (CEI) de la Universidad Peruana de Ciencias Aplicadas (UPC). Al basarse en el análisis de una base de datos de las historias clínicas de un hospital público, se procedió a anonimizar los datos que permitan el reconocimiento de los pacientes. No se discriminó ningún registro por sexo, edad, lugar de procedencia o estado socioeconómico. Debido a que la información se obtuvo de una base de datos no se requirió de consentimiento informado. El código del Registro de Proyectos de Investigación en Salud (PRISA) del Instituto Nacional de Salud de Perú fue EI00000002568.

## HALLAZGOS

A inicios del 2020 se aprecia una reducción del promedio de pacientes atendidos (Figura 1). Después de la orden del estado de emergencia y del aislamiento social obligatorio, el servicio de emergencia del HEP mostro una disminución del número de pacientes atendidos, de 669 en el 2019 a 326 en el 2020. A partir de la semana 25 se presenta un aumento gradual de las atenciones. En la semana 45 se observa un aumento con su pico máximo en la semana 47 con 970 atenciones en el 2020.

**Figura 1:** Asistencia de pacientes pediátricos por semanas en el servicio de emergencias del Hospital de Emergencias Pediátricas entre los años 2019 y 2020 (n=57 845).

En la Tabla 1 se resalta la disminución promedio del 10,2% de atenciones a preescolares en el 2020. En el caso de la procedencia de los pacientes se destaca un aumento de cinco veces más las atenciones a pacientes provenientes de otras localidades diferentes de Lima Metropolitana. Según tipo de seguro, se muestra un aumento proporcional de 3,3% de pacientes con el Seguro Integral de Salud (SIS) y una disminución proporcional del mismo valor de pacientes sin seguro.

**Tabla 1:** Características demográficas de los pacientes pediátricos atendidos durante la primera ola en el servicio de Emergencia del Hospital de Emergencias Pediátricas entre los años 2019 y 2020 (n=41 470).

Característica	Pre-COVID (2019) (n=29 736)		COVID (2020) (n=11 734)		Valor de p
	n	%	n	%	
Sexo					0,012
Femenino	13 031	43,8	5302	45,2	
Masculino	16 705	56,2	6432	54,8	
Grupo de edad					<0,001
Lactante (<12 meses)	1755	5,9	1319	11,2	
Preescolar (1 - 5 años)	19 958	67,1	6685	57,0	
Escolar (6 - 11 años)	6284	21,1	2660	22,7	
Adolescente (12 - 17 años)	1739	5,9	1070	9,1	
Procedencia					<0,001
Lima Metropolitana	28 772	96,8	9737	83,0	
Otras regiones	964	3,2	1997	17,0	
Zonificación de Lima					0,553
Norte	1227	4,3	495	4,4	
Centro	12 036	41,8	4783	42,4	
Sur	1710	5,9	679	6,0	
Este	13 799	48,0	5319	47,2	
Tipo de Seguro					<0,001
SIS	24 443	82,2	10 028	85,5	
Seguro privado	44	0,2	25	0,2	
Servicio social	35	0,1	6	0,1	
Sin seguro	5214	17,5	1675	14,3	

SIS: Seguro Integral de Salud.

Servicio social: trabajador del hospital + apoyo especial

En la Tabla 2 se observa en el 2020 un aumento del 7,5% de pacientes nuevos y una disminución promedio del 10% de pacientes continuadores en comparación con el 2019. Asimismo, en el 2020 se encontró una disminución de 4,1% en el número de altas del servicio de emergencia del HEP.

**Tabla 2:** Condición de ingreso y destino final de los pacientes pediátricos atendidos durante la primera ola en el servicio de Emergencia del Hospital de Emergencias Pediátricas entre los años 2019 y 2020 (n=41 470).

Característica	Pre-COVID (2019) (n= 29 736)		COVID (2020) (n= 11 734)		Valor de p
	n	%	n	%	
Condición al ingreso					<0,001
Nuevo	9608	32,3	4668	39,8	
Reevaluado	8261	27,8	3563	30,3	
Continuador	11 867	39,9	3503	29,9	
Destino final					<0,001
Alta	23 801	80,0	8913	75,9	
Alta + telemonitoreo	0	0	288	2,5	
Hospitalización	671	2,3	538	4,6	
Reevaluación	2620	8,8	1252	10,7	
Sala de operaciones	227	0,8	144	1,2	
Sala de observación	2324	7,8	534	4,6	
Transferencia	88	0,3	62	0,5	
Fallecido	5	0,0	3	0,0	

Reevaluación: reevaluación + interconsulta + sala de rehidratación oral + sala de asma bronquial

En la Tabla 3, al comparar los diagnósticos por código CIE-10, se encontró que en el 2020 hubo 27% mayor probabilidad de diagnóstico de «A00-B99: enfermedades infecciosas y parasitarias» en comparación con el 2019. En el caso de las «J00-J99: enfermedades del sistema respiratorio» se halló que en el 2020 hubo 61% menor probabilidad en comparación con el año 2019. Al mismo tiempo, se aprecia 80% mayor probabilidad del diagnóstico de «S00-T98: traumatismo, envenenamiento y algunas otras consecuencias de causa externa» en el 2020 con respecto al 2019. Asimismo, en el análisis del diagnóstico «K00-K93: enfermedades del sistema digestivo» se encontró una disminución en 32% en la probabilidad diagnóstico en el 2020 en comparación con el 2019. Por último, se analizó el código U00-U99, que incluía a pacientes con diagnóstico de COVID-19, donde se encontró un promedio de 21 casos semanales durante el 2020.

**Tabla 3:** Diagnóstico por código CIE-10 de los pacientes pediátricos atendidos durante la primera ola en el servicio de Emergencia del Hospital de Emergencias Pediátricas entre los años 2019 y 2020 (n=76 254).

Diagnóstico Grupo CIE-10	Pre-COVID (2019) (n = 55 801)		COVID (2020) (n = 20 453)		Diferencia porcentual %	RP crudo (IC 95%)
	n	%	n	%		
R00- R99: Síntomas, signos y hallazgos anormales clínicos y de laboratorio, no clasificados en otra parte	3902	7,0	5350	26,2	19,2	3,74 (3,60 - 3,88)
U00-U99: Códigos para situaciones especiales	0	0	790	3,9	3,9	-
N00-N99: Enfermedades del aparato genitourinario	1118	2,0	1145	5,6	3.6	2,79 (2,57 - 3,02)
C00-D48: Neoplasia	29	0,1	53	0,3	0,2	4,98 (3,17 - 7,83)
Q00-Q99: Malformaciones congénitas, deformidades y anomalías cromosómicas	217	0,4	226	1,1	0,7	2,84 (2,36 - 3,42)
F00-F99: Trastornos mentales y del comportamiento	54	0,1	63	0,3	0,2	3,18 (2,21 - 4,57)
Z00-Z99: Factores que influyen en el estado de salud y contacto con los servicios de salud	179	0,3	188	0,9	0,6	2,86 (2,33 - 3,51)
O00-O99: Embarazo, parto y puerperio	1	0,0	2	0,0	0,0	5,45 (0,49 - 60,17)
D50-D89: Enfermedades de la sangre y de los órganos hematopoyéticos y otros trastornos que afectan el mecanismo de inmunidad.	44	0,1	23	0,1	0,0	1,42 (0,86 - 2,36)
P00-P96: Ciertas afecciones originadas en el periodo perinatal	67	0,1	37	0,2	0,1	1,50 (1,00 - 2,25)
V00-Y98: Causas externas de morbilidad y de mortalidad	415	0,7	384	1,9	1,2	2,52 (2,19 - 2,89)
G00-G99: Enfermedades del sistema nervioso	206	0,4	158	0,8	0,4	2,09 (1,70 - 2,57)
M00-M99: Enfermedades del sistema osteomuscular y del tejido conectivo	201	0,4	139	0,7	0,3	1,88 (1,52 - 2,34)
H00-H59: Enfermedades del ojo y sus anexos	92	0,2	26	0,1	-0,1	0,77 (0,49 - 1,19)
L00-L99: Enfermedades de la piel y tejido subcutáneo	1437	2,6	1165	5,7	3,1	2,21 (2,05 - 2,38)
I00-I99: Enfermedades del sistema circulatorio	536	1,0	97	0,5	-0,5	0,49 (0,39 - 0,61)
E00-E90: Enfermedades endocrinas, nutricionales y metabólicas	899	1,6	299	1,5	-0,1	0,90 (0,79 - 1,03)
H60-H95: Enfermedades del oído y de la apófisis mastoides	811	1,5	149	0,7	-0,8	0,50 (0,42 - 0,59)
S00-T98: Traumatismos envenenamiento y algunas otras consecuencias de causa	3847	6,9	2551	12,5	5,6	1,80 (1,72 - 1,90)
A00-B99: Ciertas enfermedades infecciosas y parasitarias	9956	17,9	4646	22,8	4,9	1,27 (1,23 - 1,31)
K00-K93: Enfermedades del sistema digestivo	9725	17,4	2426	11,9	-5,5	0,68 (0,65 - 0,71)
J00-J99: Enfermedades del sistema respiratorio	10 488	18,8	1534	7,5	-11,3	0,39 (0,37 - 0,41)

RP: razón de prevalencia

## DISCUSIÓN

Se ha observado una reducción de 60,5% en los casos atendidos en el servicio de emergencia del HEP, posterior al inicio de la primera ola en territorio nacional; siendo mayor la disminución de atenciones en el grupo de preescolares (10,2%). Asimismo, aumentó el número de pacientes atendidos que provenían de otras regiones distintas a Lima Metropolitana (13,8%). A su vez, se encontró una disminución del 61% en la probabilidad de sufrir «enfermedades del sistema respiratorio» y un aumento del 80% en el grupo diagnóstico de «traumatismos, envenenamiento y algunas otras consecuencias de causas externa» con respecto al 2019. 

La reducción de casos es similar a la reportada en el estudio de Jang *et al*. en el cual encontraron una disminución de 62% de pacientes pediátricos en el servicio de emergencias en un hospital de Corea en el 2020 ^14^. Este resultado puede reflejar una disminución de la necesidad de usar el servicio de emergencia o relacionarse con el retraso de diagnósticos de gravedad; detallado en los reportes de Levine e Isba ^15^ ^,^ ^16^.

Como se mencionó, existe preocupación por la reducción del número de las atenciones en los pacientes pediátricos. Pepper *et al*. reportaron una disminución del 48% en las atenciones más urgentes en el servicio de emergencia pediátrica ^11^. Además, Pines *et al*. detallaron una disminución en los casos de apendicitis, septicemia e intususcepción del 19%, 49% y 42%, respectivamente, en el 2020 en comparación con el 2019 ^17^.

En el 2020 existió un aumento paulatino de las atenciones realizadas en el servicio de emergencia del HEP. Esto tendría relación con la necesidad de no cumplir con la cuarentena, puesto que no es factible para la familia promedio, el aislamiento total. Se observó un aumento de casos a partir de la semana 45, lo que coincide con movilizaciones realizadas en el país ^18^; las cuales podrían haber incrementado la transmisión de enfermedades.

Sobre el aumento de casos procedentes fuera de Lima Metropolitana, esto posiblemente se explique por una mayor aceptación de referencias desde establecimientos de salud con una menor capacidad resolutiva. Asimismo, la disminución de los pacientes sin seguro es proporcional con el aumento del número de pacientes atendidos afiliados al SIS, el cual está diseñado para la protección de los sectores más vulnerables de la población. En el 2020 hubo un aumento de pacientes nuevos y reevaluados en comparación con el año anterior; sin embargo, se observa una reducción en el número de pacientes continuadores. Una posible explicación es que estos pacientes no acudieron a sus consultas por miedo al contagio de la COVID-19.

Con respecto a los diagnósticos, el cambio más llamativo es la disminución del grupo de “enfermedades del sistema respiratorio” en el año 2020, explicada por la cuarentena y el uso de mascarillas obligatorias. En Italia, Brisca *et al*. reportaron hallazgos similares con una reducción de casos de enfermedades respiratorias en 10,8% ^12^. De la misma forma, Raucci *et al*. encontraron una disminución de 63% en patologías respiratorias debido a las medidas restrictivas y la poca exposición a alérgenos inhalados ^19^.

En el 2020, los diagnósticos del grupo de «enfermedades infecciosas y parasitarias» aumentaron, posiblemente por el contagio mediante fómites, mal lavado de manos o poca eficiencia al guardar alimentos para su posterior consumo. Un estudio realizado en Italia describió un aumento de 1,1% en enfermedades asociadas al sistema digestivo ^19^. Asimismo, con respecto al grupo de «traumatismos y envenenamiento», la bibliografía refiere una disminución de casos, puesto que existen restricciones para actividades fuera de casa ^2^ ^,^ ^19^ ^,^ ^20^. Sin embargo, se observa un aumento de diagnósticos, lo cual podría relacionarse con maltrato doméstico y negligencia durante la cuarentena ^20^.

El presente estudio posee varias limitaciones. En primer lugar, al analizar un solo establecimiento de salud, los resultados no pueden ser generalizados a la población pediátrica peruana. En segundo lugar, los diagnósticos fueron basados en el criterio del médico, que completó la historia clínica de la cual se obtuvieron los datos para la base hospitalaria analizada. Finalmente, al usar una base de datos secundaria, el análisis solo se puede realizar con los datos disponibles. Como ejemplo, no se pudo indagar los motivos de atención de los pacientes o evaluar la severidad de la enfermedad al llegar al servicio de Emergencia, por falta de información de triaje. 

Se concluye que hubo una disminución de las atenciones realizadas en el servicio de Emergencia del HEP en el 2020 en comparación con el 2019. Asimismo, se encontró un aumento de pacientes procedentes de otras regiones diferentes de Lima Metropolitana. A estos hallazgos, se añade una disminución de «enfermedades del sistema respiratorio» y un aumento de «traumatismos y envenenamientos».

Debido a estos hallazgos, los autores recomiendan analizar los factores que pudieron influir en el aumento de casos de traumatismos en el servicio de emergencia del HEP. Esta recomendación se basa en los diversos programas contra la violencia doméstica como el Programa Nacional AURORA, que cuenta con diversas estrategias para evitar esta problemática. Del mismo modo, se deben realizar diversos estudios multicéntricos para poder generalizar los resultados en la población pediátrica peruana en los años 2019 y 2020.
